# Human papillomavirus and Epstein-Barr virus infections in breast cancer from chile

**DOI:** 10.1186/1750-9378-6-7

**Published:** 2011-06-23

**Authors:** Francisco Aguayo, Noureen Khan, Chihaya Koriyama, Carolina González, Sandra Ampuero, Oslando Padilla, Luisa Solís, Yoshito Eizuru, Alejandro Corvalán, Suminori Akiba

**Affiliations:** 1 Virology Program, I.C.B.M., Faculty of Medicine, University of Chile, Independencia 1027, Santiago 838-9100, Chile; 2 Department of Public Health, Kagoshima University Graduate School of Medical and Dental Sciences, 8-35-1 Sakuragaoka, Kagoshima 890-8544, Japan; 3 Department of Public Heath, Faculty of Medicine, Pontificia Universidad Católica de Chile, Santiago, Chile; 4 Pathology Department, Faculty of Medicine, Pontificia Universidad Católica de Chile, Santiago, Chile; 5 Division of Oncogenic and Persistent Viruses, Center for Chronic Viral Diseases, Kagoshima University Graduate School of Medical and Dental Sciences, 8-35-1 Sakuragaoka, Kagoshima 890-8544, Japan; 6 Department of Hematology and Oncology, Pontificia Universidad Catolica, 85 Lira Street, Santiago 133-202, Santiago, Chile

**Keywords:** papillomavirus, breast, cancer, HPV, integration

## Abstract

**Background:**

Human papillomavirus (HPV) and Epstein Barr virus (EBV) have been found in breast carcinomas (BCs) around the world. In this study, fifty-five BCs from Chile were analyzed for HPV and EBV presence. In addition, HPV-16 viral load/physical status and E6/E7 expressions were determined.

**Results:**

The amplification of a housekeeping gene showed that 46/55 samples (84%) had amplifiable DNA. HPV-16 was detected in 4/46 BCs (8.7%) and EBV was detected in 3/46 (6.5%) BCs. The analysis of HPV-16 physical status showed that this virus was integrated in all of the tumors with a relatively low viral load (range: 0.14 to 33.8 copies/cell). E6 and E7 transcripts, however, were not detected in any HPV-16 positive specimens. Using a Cox-regression model, we found a statistically significant association between EBV presence and poor survival (p = 0.013).

**Conclusions:**

The findings in this study suggest that it is unlikely that HPV and/or EBV play a direct role in the etiology of BC.

## Background

Breast cancer (BC) is a leading cause of death in women around the world. In Chile, BC is the second cause of death by cancer with a mortality rate of 11.0/100.000 habitants [1]. It is known that only 5-10% of BCs arise in women with familial history and 90-95% are sporadic [2]. Thus, environmental factors are prominent in the etiology of this malignancy. Viral infections are associated with 20% of the cancer around the world. Only two viruses, human papillomavirus (HPV) and Epstein Barr virus (EBV) are associated with 38% of all virus-associated neoplasia [3]. In addition, these two viruses have been incriminated in the development of BC [4,5]. The HPV and EBV presence in BCs is highly variable worldwide, and their etiological role remains highly controversial. HPV is the causal agent of cervix-uterine cancer and anogenital malignancies [3]. However, HPV has been detected in extragenital tumors such as oral, esophageal, tonsillar, laryngeal, lungs and BC [5]. Di Lonardo was the first reporting HPV presence in 29.4% of BCs using polymerase chain reaction (PCR) [6]. In addition, functional studies were made in the early 90's reporting that both, HPV-16 and -18 were able to immortalize and change the proliferative properties of epithelial breast cells [7]. In another study, using a mouse model it was reported that estrogen and the loss of p53 synergized to induce mammary tumors in the presence of HPV [8]. On the other hand, EBV is associated with nasopharyngeal carcinoma and Burkitt's lymphoma, and has been involved in the development of a subset of gastric cancer and BCs [4]. Thus, the aim of this study was to analyze the presence of HPV and EBV in BC from Chile, an Asia-Pacific area country where no information about the HPV/EBV presence in BC is available. In addition, in order to shed a light about the function of HPV in this tumor, we determined the integration status, viral load and E6/E7 transcripts expression in positive cases.

## Results

**Figure 1:**
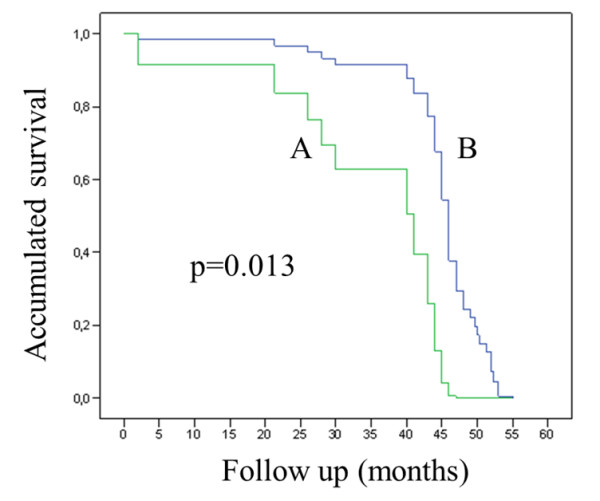
**Cox-regression survival curves**. These curves were constructed with age, number of nodes, percentage of positive nodes, histology, differentiation and tumor size. A: EBNA-1 positive women; B: EBNA-1 negative women.

In this study, HPV and EBV sequences were searched in BCs from patients living in Santiago of Chile. Fifty-five formalin-fixed paraffin-embedded BCs from different public and private hospitals were collected and the extracted DNAs analyzed for integrity by amplifying a 110 bp fragment of the betaglobin gene. Forty-six samples out of 55 were betaglobin positive (84%). Using conventional PCR for a 65 bp fragment of L1 region, HPV was detected in 4/46 (8.7%) BCs. Using qRT-PCR for EBV EBNA-1, EBV was present in 3/46 (6.5%) BCs. Co-infection with both viruses was only detected in 1/46 (2.1%) cases. In addition, ISH for EBER-1 showed that all of the BCs were EBV negative. A non-statistically significant association between HPV presence and age, tumor size, histology and differentiation grade was found (p = 1.000; 0.780; 1.000 and 0.721 respectively, Table 1). HPV infection was more frequently detected in primary tumors from patients with metastatic lymph nodes, although it was not statistically significant (p = 0.078). On the other hand, no statistically significant association between EBV presence and age, lymph nodes involvement, tumor size, histology and differentiation grade was found (p = 1.000, 1.000, 0.459, 0.130 and 0.255, respectively, Table 1). Using a Cox-regression model, a statistically significant association between EBNA-1 positivity by qRT-PCR and poor survival was found (p = 0.013, Figure 1). The Inno-Lipa method showed that only the HPV-16 genotype was present in HPV positive BCs without co-infections with other HPV genotypes. In addition, the number of copies of HPV-16 determined by real-time PCR was extremely low in 1/4 cases studied (0.14 copies/cell). In the other three cases, the viral load was higher (1.2; 2.0 and 33.8 copies/cell, Table 2). The absence of E2 amplification showed that HPV-16 was completely integrated into the host genome in all of the cases. In addition, we were unable to detect HPV16 E6 and E7 transcripts in 3/3 HPV-16 positive cases as showed using transcriptase-reverse real-time PCR, even though the specimens were positive for β2-microglobulin expression (Table 2).

**Table 1:** Relationship between clinicopathological features of breast carcinomas and HPV/EBV presence.

			HPV (L1)			EBV (EBNA-1)		
				
		All	HPV+	HPV-	p-value	EBV+	EBV-	p-value
		**N (%)**	**N(%)**	**N(%)**		**N(%)**	**N(%)**	

Age					1.000			1.000
	< 65 years	34	3 (9)	31 (91)		2 (6)	32 (94)	
	> 65 years	12	1 (8)	11 (92)		1 (8)	11 (92)	
Lymph nodes					0.078			1.000
	negative	32	1 (3)	31 (97)		2 (6)	30 (94)	
	positive	14	3 (21)	11 (79)		1 (7)	13 (93)	
Tumor size					0.780			0.459
	< 2 cm	7	0 (0)	7 (100)		1 (14)	6 (86)	
	2-5 cm	31	4 (13)	27 (87)		2 (6)	29 (94)	
	> 5 cm	8	0 (0)	8 (100)		0 (0)	8 (100)	
Histology					1.000			0.130
	ductal	42	4 (10)	38 (90)		2 (5)	40 (95)	
	lobulillar	1	0 (0)	1 (100)		1 (100)	0 (0)	
	duct/lob	2	0 (0)	2 (100)		0 (0)	2 (100)	
	mucinoso	1	0 (0)	1 (100)		0 (0)	1 (100)	
Differentiation					0.721			0.255
	well	3	0 (0)	3 (100)		1 (33)	2 (67)	
	moderately	17	1 (6)	16 (94)		1 (6)	16 (94)	
	poor	23	3 (13)	20 (87)		1 (4)	22 (96)	
	unknown	3	0 (0)	3 (100)		0 (0)	3 (100)	

**Table 2:** Physical status, viral load and E6/E7 expression in HPV-16 positive breast carcinomas

Sample	E6 (copies/cell)	E2 (copies/cell)	E2/E6	Physical status	E6/E7 transcripts
BC2	2	0	0	integrated	negative
BC21	33,8	0	0	integrated	ND^a^
BC31	1,2	0	0	integrated	negative
BC5	0,14	0	0	integrated	negative

^a^ND: non-determined (specimen exhausted)

## Discussion

The etiological role of HPV or EBV in some extragenital tumors is under intense debate. In this report, the presence of HPV and EBV was evaluated in BCs from Chile, a Latin American country from the Pacific Ocean area. Our findings showed that HPV and EBV prevalence in BCs was relatively low since these viruses were present in 8.7% and 6.5% of breast tumors, respectively. The high-risk HPV-16, the genotype most frequently detected in cervix uterine cancer and extragenital malignancies in Chile and in the world [9,10] was the only genotype identified in HPV positive BCs. However, the presence of HPV genomes is not a sufficient condition to establish a causal relationship. For this reason, we investigated the physical status of HPV and the presence of E6/E7 transcripts into the BC tumors. Thus, we found that HPV-16 was integrated into the host genome in all of the cases.

Other authors have analyzed HPV in BC specimens, suggesting a causal association. Hennig et al. proposed that HPV can be transported by circulation (bloodstream/lymphatic system) from cervix to the breast [11]. In addition, it has been reported the presence of HPV-6/11 and other papillomaviruses in nipple and tumoral tissue from patients with BC, even though high-risk HPV was present in a low proportion of cases [12]. In Asia, it has been reported a 20% of high-risk HPV in breast tumors from Japanese women and HPV-16 was the most frequent HPV genotype that was integrated into the host genome with a low viral load [13]. In Latin America, only three studies have been reported: in Brazil it was reported HPV-16/18 presence in 24.8% of BCs [14] and recently it was published that 79 analyzed BCs were negative for HPV-6, 11, 16 and 18 [15]. In Mexico, HPV was found in 29.4% of BC, being HPV-16 the most prevalent genotype [16]. However, in the same country, HPV was detected in 10% of 60 BCs [17]. In this last study, HPV was found integrated with a low viral load. In Oceania (Australia), it has been reported a study using *In situ *PCR for the successful detection of HPV in BCs. High-risk HPV was detected in the nucleus of 11/43 (25.6%) BCs [18]. On the other hand, HPV-18 genotype was frequently found in Australian BCs [19].

The loss of E2 gene function by HPV integration is known to be one of the major genetic events facilitating transformation and transition to malignancy [9]. The E2 loss allows abnormal expression of E6 and E7 oncoproteins [20]. The mechanism of high-risk HPV-mediated oncogenesis involves the interaction of E6 oncoprotein with the p53 tumor suppressor protein and the E6-associated protein for induction of proteasomal-dependant p53 degradation [3]. In addition, the high-risk HPV E7 binding to pRb protein induces E2F releasing and overexpression of p16^INK4a ^by a negative feedback mechanism [21]. High-risk HPVs are frequently integrated in high-grade cervical intraepithelial neoplasia lesions and low-risk HPVs are frequently present in an episomal form in low-grade or benign lesions [22]. The analytical approach used in this report, considers HPV-16 integrated when E2 gene is not amplified using quantitative real-time PCR. This is an indirect method, based in the frequent E2 disruption when HPV is integrated into the host genome. However, HPV is able to integrate disrupting other regions as E1 [23]. So, when E2 is successfully amplified is not possible discard HPV-16 integration. However, we were unable to amplify a fragment of E2 gene in four cases that previously were positive for HPV-16 presence. Thus, E2 loss by integration is the biological possibility that explains these results.

Even though, HPV-16 was present and integrated in BCs, we were unable to detect E6 and E7 transcript expressions in the specimens. The successful amplification of a fragment of β2-microglobulin was used as control of cDNA preparation and amplification from paraffin-embedded tissues. It has been reported that in general HPV load in extragenital tumors is heterogeneous and lower that in cervical cancer [13,24,25] so it was expected that E6/E7 transcript levels were very low too, in concordance with the viral load. However, the absence of detectable levels of E6/E7 transcripts is a hallmark of absence of functional activity in HPV-16 positive specimens. The RNA obtained from paraffin-embedded cervical carcinomas was used as control, and we were able to detect E6/E7 transcripts in all of them (data not shown). Curiously, this is the first study that addresses the expression of E6 and E7 transcripts in breast carcinomas, so we cannot compare our results with any previous published report. On the other hand, the viral load in three specimens (higher than unity) is compatible with an eventual direct carcinogenic role of HPV, although the absence of E6/E7 expression casts doubts about this notion. To this respect, it is possible to speculate the possibility of a "hit and run" mechanism of HPV action, however if HPV is related to the initiation, promotion or progression of breast carcinogenesis is unknown. Because PCR method is unable to determine if HPV localizes specifically in the tumoral tissue, the detected viral load in 3/4 cases allow us to speculate that at least tumoral cells are positive for HPV-16. Anyway, we cannot deny the possibility that HPV infection occurred after clonal expansion and cancer development, involving both tumoral and non-tumoral tissue. In addition, it is known that one HPV genome per cell is enough for neoplastic transformation as occur in SiHa cells harboring 1-2 HPV-16 copies/cell. Around the world, the HPV prevalence in BCs has been shown to be highly variable, ranging from 0 to 86% [26]. This difference may be explained by variations in the methodological approaches used to detect HPV. Moreover, PCR protocols showing a diverse sensitivity and specificity are currently used to determine HPV presence in tumoral tissue and a "gold standard" protocol has not been defined. However, our analytical approach has been widely used by us and was previously reported by others [13].

On the other hand, there is a relative consensus that for detection of EBV latent infection, the "gold standard" method is ISH for EBER-1 [27]. Interestingly, we detected three positive specimens using qRT-PCR for EBNA-1 and using ISH for EBER-1, the cases were negative. This discrepancy might be caused by differences in sensitivity of these methods or very low levels of EBER-1 expression in EBV infected cells. EBERs are necessary for the maintenance of malignant phenotypes of B lymphocyte cells but is unknown if are ever expressed in BC cells [28]. Recently, it has been reported a 55% of EBV presence in BCs from India [29]. Interestingly, the authors evaluated EBNA-1 expression using immunohistochemistry (IHC) and they compared with serology in women with cancer and controls. Unfortunately, the authors did not compare their results with EBER-1 detection in the same specimens. In BC, since the first study in 1993 reporting no detection of EBV in BCs [30], EBV presence has been consistently reported in a plethora of other studies [29,31-36]. However, EBV has not been detected in other reports around the world [37-40]. In this study, using a Cox-regression model we found a statistically significant association between EBV EBNA-1 positivity and poor survival. Even though the sample size was low, this data is in agreement with a previous report where a relationship between poor survival and EBV presence was found [41]. The molecular mechanism involved in poor survival in EBV- associated BC remains to be investigated. It is necessary to appoint that is not strictly necessary that a virus be directly involved as etiological agent to produce molecular alterations in the behavior of some tumor or progression of the disease. In fact, HCMV, another persistent virus, has been detected in some tumors where the virus is able to alter the host response, specifically through immune response modulation [42]. However, how HPV or EBV may potentially be involved in tumor modulation and consequently are associated with outcome or prognostic warrants more investigation.

In conclusion, we reported here that HPV and EBV prevalence in breast cancer from Chile is relatively low, thus a possible direct etiological role of these virus is unlikely. However, additional studies are warranted to elucidate the function of HPV/EBV in a subset of breast carcinomas from Chile.

## Methods

### Study subjects

Fifty-five BCs from patients of different Hospitals in Santiago of Chile were considered for this study. The Hospitals were Las Condes Clinic; San Juan de Dios Hospital; Instituto Nacional del Cáncer; San Borja Arriaran Hospital; José Joaquín Aguirre Hospital; Arturo Lopez Perez Foundation and Santa María Clinic. The average age of the patients was 58.7 years (range: 48.1-69.3). The histological types of BC were: 48 ductal, 6 lobulillar and 1 mucinous. In the present study, histological classification was made using the guidelines of the Japan Cancer Society [43], which follows the WHO classification. The Grupo Oncológico Cooperativo Chileno de Investigación (GOCCHI) approved this study.

### DNA extraction, HPV detection and genotyping

Sections of 10 μm thickness were cut and collected in sterile tubes. For DNA extraction, each sample was treated with 1 mL of xylene and subsequently washed with 1 mL of ethanol. The sediment was resuspended in 50 mM Tris-Cl pH 8.0, 1 mM EDTA, pH 8.0, and 0.5% Tween 20 containing 200 μgmL^-1 ^of proteinase K (Invitrogen Corp., Carlsbad, CA, USA) and incubated overnight at 56°C. The solution was heated at 100°C for 10 min and subsequently the DNA was purified by phenol-chloroform extraction and precipitated with cold ethanol. DNA quality was tested by PCR for betaglobin using PCO3 5'-ACACAACTGTGTTCACTAGC-3' and PCO4 5'-CAACTTCATCCACGTTCACC-3' primers under the following PCR conditions: denaturation at 95°C for 15 min, 40 cycles with the cycling profile of 95°C for 1 min, 52°C for 1 min, 72°C for 1 min and final extension at 72°C for 5 min. To detect HPV genomes, broad-spectrum SPF10-biotinylated primers were used. The PCR products, 65 bp of the L1 gene, were characterized on a 4% agarose gel and visualized under UV radiation with ethidium bromide staining. HPV genotyping was performed using the Inno-Lipa HPV Genotyping V2 test (Innogenetics, Ghent, Belgium) [44]. As negative controls, paraffin sections without tissue and distilled water were used for procedures of DNA extraction and PCR, respectively. Full genomes of HPV-16 and HPV-18 cloned in pUC19 plasmid (kindly given by Dr. Massimo Tommasino, IARC, Lyon, France) were used as positive controls for HPV amplification.

### Quantitative real-time PCR (qRT-PCR)

To determine the presence, physical status and viral load of HPV-16, real-time PCR was performed with the ABI Prism 7700 Sequence Detection System (Applied Biosystems, Foster City, CA, USA) and 2 × QuantiTect SYBR Green PCR kit (Qiagen, Hilden, Germany). The HPV-16 primers for E6 ORF amplification were as follows: E6F: 5'-GAGAAACTGCAATGTTTCAGGACC-3' and E6R: 5'-TGTATAGTTGTTTGCAGCTCTGTGC-3'. The HPV-16 primers for E2 ORF amplification were as follows: E2F: 5'-AACGAAGTATCCTCTCCTGAAATTATTAG-3' and E2R: 5'-CCAAGGCGACGGCTTTG-3'. The PCR conditions were 2 min at 50°C, 15 min at 95°C, and a two-step cycle of 95°C for 15 s, and 60°C for 60 s for a total of 40 cycles [45]. The sizes of the E6 and E2 products were 81 and 76 bp, respectively. Dilutions of the full-length HPV-16 genome cloned in pBR-322 vector, from 86 to 862 million copies per reaction, served as a standard for calibration curves for E2 and E6 genes. Linear plots of the log of copy number vs. numbers of threshold cycle were consistently obtained for both genes. As positive control, the cell line SiHa containing 1 copy of HPV-16 per cell was used. Real-time PCR for a betaglobin gene fragment was performed by the 2 × QuantiTect SYBR Green PCR kit (Qiagen) using PC03/PC04 to adjust the differences in the amount of input genomic DNA between samples. A seven-fold dilution series of a human DNA control (Dynal, UK Ltd, Bromborough, Wirral, Merseyside, UK) was used to generate the standard curve. The amount of betaglobin DNA present in each sample was divided by the weight of one genome equivalent (that is, 6.6 pg per cell) and a factor of 2 (as there are two copies of β-globin DNA/genome equivalent or cell) to obtain the number of genome equivalents. For EBV detection, the PCR conditions were 2 min at 50°C, 15 min at 95°C, and a two-step cycle of 95°C for 15 s and 60°C for 60 s for a total of 40 cycles. The primers sequence was as follows: EBV-F 5'-TACAGGACCTGGAAATGGCC-3' and EBV-R: 5'-TCTTTGAGGTCCACTGCCG-3'. The amplified fragments in positive specimens were characterized by melting analysis and checked by agarose gel electrophoresis.

### qRT-PCR for HPV-16 E6 and E7 transcripts

The RNA purification was carrying out using the High Pure RNA paraffin kit (Roche), according to instructions of the manufacturer. The obtained RNA was resuspended in 50 μL of TE (10 mM Tris-Cl, 1 mM EDTA) and stored at -80°C until use. The cDNA preparation was made as follows: 100 ng of purified RNA were transformed to cDNA using RNAsin 1 U/μL (Promega, USA); 1× buffer TR (Promega, USA); 10 μg/μL random primers (Promega, USA); 20 U/μL MMLV (Promega, USA) and 2 mM dNTPs in a final volume of 20 μL. The reaction mixture was incubated at 37°C for 1 h, 70°C for 15 min and stored at -20°C. Ten microliters of cDNA were used for qRT-PCR. The amplification reaction was carrying out separately for HPV-16 E6 and E7 transcripts using the SensimixSYBR (Bioline) kit in a final volume of 25 μL. For E6, the reaction mixture was composed by 1X SensiMix, 0.8 μM primers and 5 mM MgCl_2_. The conditions of amplification were: denaturation at 95°C for 10 min, 45 cycles of 95°C for 15 s, 55°C for 20 s, 72°C for 20 s and a final extension at 72°C for 20 s. The sequence of primers was: E6F: 5'-CAACAAACCGTTGTGTGAT-3'; E6R: 5'-CGTGTTCTTGATGATCTGC-3'. A melting curve analysis between 62°C to 95°C with a variation of 0.5°C was made. The melting temperature (Tm) of the amplification product from a positive control was 79.85°C. For E7, the reaction mixture was composed by 1X SensiMix, 0.8 μM primers and 3 mM MgCl_2_. The conditions of amplification were initial denaturation at 95°C for 10 min followed by 45 cycles consisting of denaturation at 95°C for 15 s, annealing at 57°C for 20 s and extension at 72°C for 20 s. The sequence of primers was E7F: ATGCATGGAGATACACCTAC; E7R: 5'-CATTAACAGGTCTTCCAAAG-3'. The amplification of β2-microbulin gene was carrying out as follow: 0.8 μM Primer F; 0.8 μM primer R, 5 mM MgCl_2_, 1X Sensimix and 50 ng cDNA. The amplification program was initial denaturation at 95°C for 10 min, followed by 45 cycles consisting of denaturation at 95°C for 15 s, annealing at 55°C for 20 s and extension at 72°C for 20 s. The sequence of primers was: B2MF: 5'-TGTAAGCAGCATCATGGA-3' and B2MR: 5'-AGTGTAAGTGTATAAGCATATCAA-3'. The melting point of the amplified product was 81.15°C. All the amplifications were made using Rotor Gene 6000 (Corbett Research) real-time PCR equipment.

### Tissue microarray (TMA) construction

Fifty-seven specimens of BC were used to construct a tissue array as previously described [46]. The tumoral area of the clinical specimens was selected by experienced pathologists (LS, AC).

### In situ hybridization (ISH) for EBV

In situ hybridization (ISH) with a complementary digoxigenin-labeled 30-base probe was used to detect EBER-1 expression according to the procedure previously published [27]. Briefly, 4-5-μm sections mounted on silane-coated glass slides were prepared. The tissue on the slides (tissue array) was deparaffinized, rehydrated, predigested with Pronase, prehybridized, and then hybridized overnight at 37°C with 0.5 ng digoxigenin-labeled probes. The hybridization signal was detected by an anti-digoxigenin antibody-alkaline phosphatase conjugate. Sections from a patient with known EBER-positive gastric carcinoma were used as positive controls, and sense probe to EBER-1 was used as negative control.

### Statistical analysis

Fisher's exact test was used for determine differences between groups. P-value < 0.05 was considered statistically significant. For survival analysis, a Cox-regression model was used.

## Competing interests

The authors declare that they have no competing interests.

## Authors' contributions

FA, SAk, AC and CK conceived of the study, analyzed the data and participated in the redaction of the manuscript. CG and NK made the analysis of the clinical specimens and analyzed the data. AC and LS got specimens for analysis, gave clinical information, and gave clinicopathologic support. OP gave statistical support and analysis. YE, CK and SAm gave analytical support for analysis. All authors read and approved the final manuscript.
